# A global definition of expression context is conserved between orthologs, but does not correlate with sequence conservation

**DOI:** 10.1186/1471-2164-7-10

**Published:** 2006-01-19

**Authors:** Bas E Dutilh, Martijn A Huynen, Berend Snel

**Affiliations:** 1 Center for Molecular and Biomolecular Informatics / Nijmegen Center for Molecular Life Sciences, Radboud University Nijmegen. Toernooiveld 1, 6525 ED, Nijmegen, The Netherlands

## Abstract

**Background:**

The massive scale of microarray derived gene expression data allows for a global view of cellular function. Thus far, comparative studies of gene expression between species have been based on the level of expression of the gene across corresponding tissues, or on the co-expression of the gene with another gene.

**Results:**

To compare gene expression between distant species on a global scale, we introduce the "expression context". The expression context of a gene is based on the co-expression with all other genes that have unambiguous counterparts in both genomes. Employing this new measure, we show 1) that the expression context is largely conserved between orthologs, and 2) that sequence identity shows little correlation with expression context conservation after gene duplication and speciation.

**Conclusion:**

This means that the degree of sequence identity has a limited predictive quality for differential expression context conservation between orthologs, and thus presumably also for other facets of gene function.

## Background

The two main components of the function of a gene are its molecular function (what does it do, e.g. is it a hydrolase, is it DNA binding) and its functional context (with what other elements of the cell does it collaborate). Though both aspects can only be decisively determined in *in vivo *experiments, the incredible and increasing amount of experimental information assembled in databases enables more and more accurate predictions [1]. Because of the accuracy and speed with which algorithms can identify sequence similarity, the most commonly used tool for predicting gene function is doubtlessly sequence conservation. As the sequence is the blueprint for the three-dimensional structure, and therewith the enzymatic function of a gene, this method is particularly suitable for predicting the molecular function of an unknown gene, for example in a newly sequenced species.

Predicting functional context, on the other hand, is a different story. This means inferring *in silico *in which process the gene plays a role. Whereas the molecular function is concrete, and can be described by the catalyzed chemical reaction, the functional context is more elusive and may best be described as a composition of the context (e.g. binding partners) of the encoded protein and the regulation of its expression in time and space [2]. A way to estimate the functional context is in terms of the collection of cells or tissues and biological processes or circumstances that determine when the gene is expressed. DNA microarrays measure the expression levels of many genes under the same experimental condition, and combining the information from many such experiments allows the clustering of genes based on correlations in their expression patterns [3]. If two genes are co-expressed, i.e. they have a comparable expression profile, they are assumed to have a comparable functional context, independent of what this functional context is. Using co-expression as a function prediction tool is particularly powerful when the co-expression is conserved in different organisms [4-7].

Here, we introduce a method to take the step from the comparative study of expression evolution based on the pairwise co-expression between two genes, to a definition on a global level. We present the "expression context" of a gene, based not on the expression across a range of tissues or circumstances, but on the co-expression with a range of genes. If two genes are co-expressed with the same other genes, i.e. they have a comparable co-expression profile, they thus have a comparable expression context. Not only does this allow a global view on expression evolution, but it also solves the issue of comparing gene expression between distantly related species. When studying e.g. *Caenorhabditis elegans *and *Saccharomyces cerevisiae *[5], one can not assign equivalent tissues like between *Homo sapiens *and *Mus musculus *[8]. The expression context method overcomes this limitation by substituting identical tissues for orthologous genes, and levels of expression for co-expression values. In this study, we include four Eukaryote species (*C. elegans*, *Drosophila melanogaster*, *H. sapiens *and *S. cerevisiae*), for which gene co-expression data have been determined on a large scale [6]. The first issue we address in this paper is how much our new global estimate of expression context is conserved between species.

In a comparative analysis of gene properties between different species, a solid definition of orthology is critical. Current state of the art orthology methods allow for the expansion of an orthologous gene pair in one or both of the species compared. The existence of these so called in-paralogs, raises the question to what extent the expression contexts of the gene copies have diverged. Previously, we have studied genes that are duplicated in *C. elegans *relative to *S. cerevisiae *[7]. We showed that the *C. elegans *orthologs of genes that in *S. cerevisiae *are reliably co-regulated with the ancestral gene, have a tendency to retain co-expression with one of the two duplicated orthologs in *C. elegans*, while the link with the other is lost (partial conservation, Fig. 3 in [7]). One of the important questions this paper left us with is whether the derived gene that had retained the ancestral regulatory context was also the least diverged at the sequence level. Therefore, the second issue addressed in the current work is the relationship between the evolution of the gene sequence and the evolution of the expression context after a gene duplication. We present an analysis between orthologous groups (after speciation), and an analysis between sibling genes (in-paralogs) within expanded orthologous groups (after gene duplication), and show that sequence and expression context tend to diverge independently.

## Results and discussion

### Orthology

Inparanoid is a pairwise definition of orthology that allows for species specific gene expansions (in-paralogs, [9]). In the case of this group orthology, two or more genes from one species are evolutionarily equally orthologous to one or more genes in the other species. Such a scheme is necessary if we want to study the divergence in expression context between two recent gene copies, which would not be found in, for example, a reciprocal best hit approach. On the other hand, algorithms that identify group orthology between more organisms at once would annul the resolution obtained in a pairwise definition [10]. We constructed orthology relationships separately for all species pairs, and separated the resulting orthologous groups into two categories: 1-1 orthologous groups (if both species contain a single ortholog) and X-X orthologs (if at least one of the species contains more than one ortholog). There are about twice as many 1-1 orthologs as there are X-X orthologous groups (see Table 1).

### Expression context

**Figure 1:**
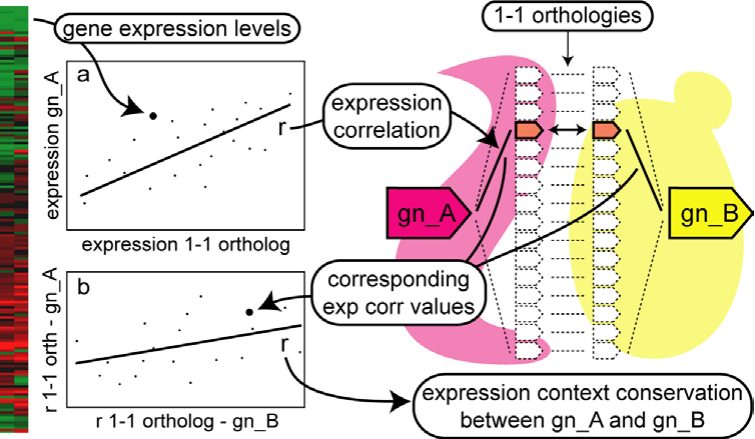
Method used to calculate the expression context conservation between gn_A and gn_B. Genes gn_A and gn_B are the query genes in species A and species B, respectively. First, the correlation between the expression levels of the query gene and all 1-1 orthologs over multiple microarray experiments was calculated in both species (a; uncentered correlation). The resulting expression correlation values were correlated between the two species (b; Pearson's correlation), yielding the expression context conservation between gn_A and gn_B. For an unambiguous comparison between species, we only analyze the expression correlation values of the studied genes with the 1-1 orthologs.

**Figure 2:**
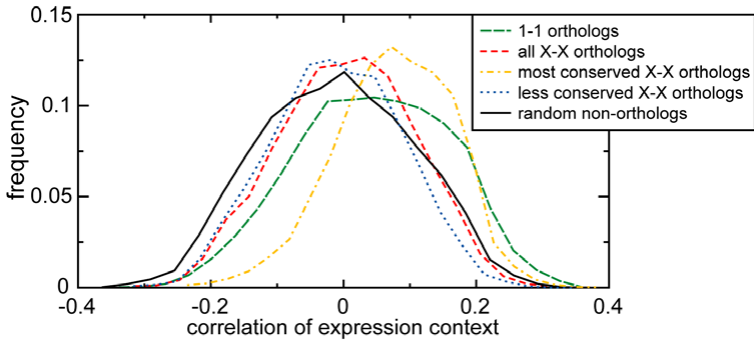
Expression context conservation between different classes of orthologs and random non-orthologous gene pairs. The plots are normalized histograms of the combined data from all species comparisons. For statistical comparison of the histograms see Table 4. The distributions are normally distributed (Shapiro-Wilk test, P < 1·10^-4^).

The global definition of expression context introduced here is based on the expression correlations between a query gene in one species and all the members in that species of all 1-1 orthologous groups present between the two species compared (see Fig. 1a). The expression context conservation is then obtained by correlating the expression correlation values of the query genes from two different species and the corresponding 1-1 orthologs in their species (see Fig. 1b). To test how meaningful this measure is, we compared the expression context conservation between different categories of orthologs and random non-orthologous gene pairs. The histograms in Fig. 2 are normalized, and the data is pooled over all species comparisons. As a null model, we composed a random data set of 1000 non-orthologous gene pairs drawn from each species pair. Though the distributions of the expression context conservation scores lie close to zero, we find that the expression context of both 1-1 orthologs and of X-X orthologs is significantly higher than that of random genes (see Fig. 2, for Pvalues see Table 2). This significant conservation reveals the functional and evolutionary relevance of the expression context.

### Which genes have a conserved expression context?

**Figure 3:**
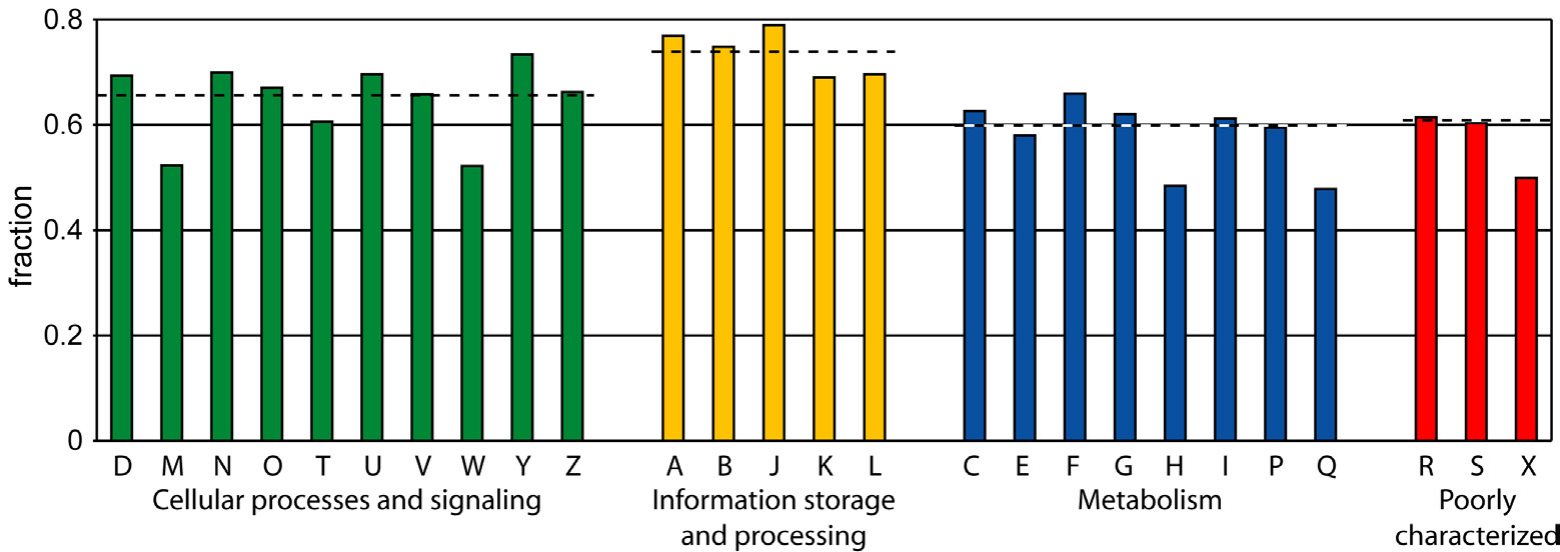
Functional classification of 1-1 orthologs with a conserved expression context (score higher than zero). From all species pairs, all 1-1 orthologs that could be assigned to a KOG were included. The categories are grouped in the four main KOG classes. The horizontal dashed lines are the fraction of genes with a conserved expression context for the entire class. The functional categories are (the number between brackets is the number of genes with a conserved expression context): "Cellular processes and signaling" (D: Cell cycle control, cell division, chromosome partitioning (n = 442), M: Cell wall/membrane/envelope biogenesis (n = 73), N: Cell motility (n = 23), O: Posttranslational modification, protein turnover, chaperones (n = 1330), T: Signal transduction mechanisms (n = 1151), U: Intracellular trafficking, secretion, and vesicular transport (n = 953), V: Defense mechanisms (n = 67), W: Extracellular structures (n = 111), Y: Nuclear structure (n = 96), and Z: Cytoskeleton (n = 378)), "Information storage and processing" (A: RNA processing and modification (n = 823), B: Chromatin structure and dynamics (n = 244), J: Translation, ribosomal structure and biogenesis (n = 1153), K: Transcription (n = 985), and L: Replication, recombination and repair (n = 545)), "Metabolism" (C: Energy production and conversion (n = 486), E: Amino acid transport and metabolism (n = 367), F: Nucleotide transport and metabolism (n = 205), G: Carbohydrate transport and metabolism (n = 452), H: Coenzyme transport and metabolism (n = 131), I: Lipid transport and metabolism (n = 383), P: Inorganic ion transport and metabolism (n = 228), and Q: Secondary metabolites biosynthesis, transport and catabolism (n = 71)) and "Poorly characterized" (R: General function prediction only (n = 1716), S: Function unknown (n = 912), and X: Not categorized by NCBI staff (n = 2)) [10].

We looked at the function of the genes with a conserved expression context using the KOG functional categories [10]. The functional categories were counted for all 1-1 orthologs assigned to a KOG (the genes were considered separately). For each functional category, the fraction of 1-1 orthologous genes with an expression context conservation score higher than zero is shown in Fig. 3. We find that all "Information storage and processing" categories have a higher level of expression context conservation than all "Metabolism" categories. Within the "Cellular processes and signaling" class, which lies between the two extremes, we also find the categories with more informational genes to have a higher expression context conservation than those containing operational genes. "Nuclear structure" (Y) for example has a large fraction of genes with a highly conserved expression context, while "Cell wall/membrane/envelope biogenesis" (M) and "Extracellular structures" (W) have a low expression context conservation. These results are in accordance with other studies: the conservation of co-expression has previously been shown to be high for genes involved in core informational cellular processes (specifically the ribosome and ribosome biogenesis [6], as well as the GO biological process category "Metabolism", which harbors protein biosynthesis [11]). Informational genes are also found to be more conserved than operational genes with respect to other properties, e.g. they have been shown to be less prone to horizontal gene transfer [12,13].

### Differential expression context conservation between in-paralogs

**Figure 4:**
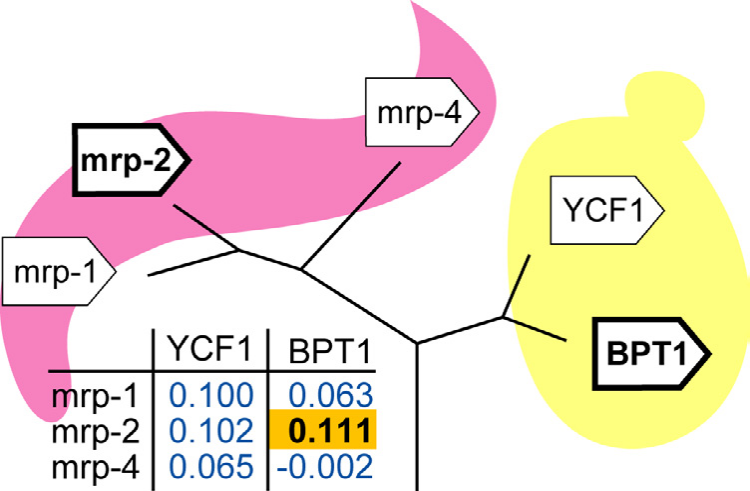
Example of an X-X orthologous group between *C. elegans *and *S. cerevisiae*. This X-X orthologous group (KOG0054: Multidrug resistance-associated protein/mitoxantrone resistance protein, ABC superfamily) has three genes in *C. elegans *and two genes in *S. cerevisiae*. The expression context conservation scores are given in the table. The gene pair with the highest score is the "most conserved X-X orthologous gene pair" (bold, yellow), the rest are the "less conserved X-X orthologs" (blue).

Our previous work suggests that in an X-X orthologous group, the ancestral expression context may have been retained by one of the in-paralogs in each of the species [7], possibly because they are functionally the most conserved. We therefore sub-classify each X-X orthologous group into the gene pair that has the highest expression context conservation within this orthologous group on the one hand (we will refer to this gene pair as the "most conserved X-X orthologous gene pair"), and on the other hand the remaining, "less conserved X-X orthologs" (Fig. 4).

Comparing the distribution of the expression context conservation scores in these sub-categories of orthologs with the other histograms in Fig. 2 reveals that only the set of random gene pairs and the less conserved X-X orthologs do not have significantly different distributions (P = 0.172, Student's t-test; see Table 2). The expression context conservation in these two data sets was lowest, followed by, in order, all X-X orthologs, the 1-1 orthologs, and finally the most conserved X-X orthologs (see Fig. 2). All the other pairs of distributions are highly significantly different from one another (P ≤ 3.55·10^-21^, see Table 2).

### Correlation of sequence identity and expression context conservation between orthologous groups

To find out how the conservation of expression context (see Fig. 2) is reflected in the sequence conservation, we first analyzed how the sequence divergence between orthologous groups relates to the divergence in expression context in an orthologous gene pair after speciation. To avoid having to make a potentially controversial choice on how to functionally and evolutionary interpret the multiple orthologous relationships in X-X orthologous groups [7], we only used the 1-1 orthologs for this comparison. These gene pairs originated at the speciation event, so they have all had the same amount of time to diverge. Table 3 presents the correlation coefficients between expression context conservation and sequence identity of the 1-1 orthologs for all species pairs.

Though the correlation coefficients are significantly positive (P < 0.05 for all species comparisons except DM-SC, where P = 0.09), they are very low (see Table 3). In this analysis of the relationship between expression context conservation and sequence identity across orthologous groups, we conclude that the evolution rate of the gene sequence does not depend on its expression context.

A trend that we seem to observe is that the correlation between sequence evolution and expression context evolution reflects the predictive span of the expression data. In Figs. 2d-f of the paper by Stuart et al.(2003), the accuracy-coverage plots of *D. melanogaster *and *H. sapiens *are always lower than those of *C. elegans *and *S. cerevisiae*. In our results, we also observe the highest correlation between expression context conservation and sequence identity for the 1-1 orthologs of *S. cerevisiae *and *C. elegans*, rather than for two closer related Metazoa. Thus some of the variation in our results reflect the quality of the microarray data for function prediction.

### Correlation of sequence identity and expression context conservation between orthologs after a single gene duplication

**Figure 5:**
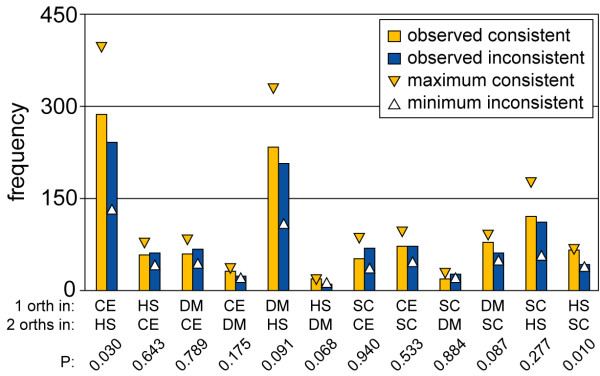
Consistency of sequence divergence with divergence in expression context for simple duplications. Consistency or inconsistency of sequence divergence with divergence in expression context for orthologous groups with a single gene duplication (1–2 orthologs). We display both the observed frequencies (plotted are the number of 1–2 orthologous groups; P is the probability to find at least this number of consistent observations by chance, binomial distribution) and the maximum consistent and minimum inconsistent frequencies expected (horizontal edge of the triangles), based on a completely consistent re-allocation of the expression context conservation scores from the overlapping distributions (see Methods).

The simplest case where we can study the divergence of duplicated genes within orthologous groups is for 1–2 orthologs, where one gene duplication occurred in one of the two daughter species since the speciation event. We carry out a straightforward analysis by counting how often the gene with the highest expression context conservation also has the highest sequence identity. Fig. 5 shows the consistency of sequence evolution with expression context evolution in the 1–2 orthologous groups.

It is immediately striking how little difference there is between the observed consistent and observed inconsistent bars in Fig. 5. For all species comparisons, there is no significant over-representation of consistent observations, apart for a few exceptions (CE1-HS2 orthologs (i.e. 1 ortholog in *C. elegans *and 2 orthologs in *H. sapiens*, other abbreviations are composed similarly) and HS1-SC2 orthologs; P < 0.05, binomial distribution). In general, all the Pvalues are very high, so this analysis shows that for 1–2 orthologs, the expression context is not better conserved in the ortholog with the highest sequence identity.

Given the large overlap between the expression context conservation scores of the most conserved X-X orthologous gene pair and the less conserved X-X orthologs (see Fig. 2), a substantial fraction of inconsistent cases is expected based on this overlap alone. We therefore examined whether the small differences between the observed consistent and inconsistent frequencies in Fig. 5 resulted from this overlap. To do this, we split the expression context conservation scores of all 1–2 orthologous groups into two data sets: one containing the highest (most conserved) expression context conservation scores, the other containing the lower (less conserved) scores. We computed the expected maximum consistent and minimum inconsistent observations by drawing from these data sets consistently with the sequence conservation (see Methods). The triangles in Fig. 5 show that many more consistent observations are expected if the data was initially organized consistently, even when the distributions of the most conserved and the less conserved X-X orthologs have such a large overlap.

In this analysis, we observed that the difference in sequence identity for the two duplicated genes was often small. This may in part be due to the fact that we compare evolutionarily divergent species, where the differences between in-paralogs (within species) are small relative to the differences between orthologs (between species). To be able to compare the rate of sequence evolution more accurately, we studied in detail the CE1-SC2 orthologous groups, and included the genome of *Ashbya gossypii*, a fungus closely related to *S. cerevisiae*. Where we found an AG1-SC2 orthologous group consisting of the same two *S. cerevisiae *genes as in the accompanying CE1-SC2 orthologous group, we calculated the K_a_/K_s _ratio between both gene pairs in the AG1-SC2 orthologous group to determine the rate of evolution for both *S. cerevisiae *genes. The ratio of nonsynonymous (K_a_) to synonymous (K_s_) nucleotide substitution rates is an indicator of selective pressures on genes [14]: a ratio higher than one indicates genes that are under positive selection pressure to change their sequence, a ratio lower than one indicates stabilizing selection. We found that the expression context was conserved for the slowest evolving *S. cerevisiae *gene in no more than 50% of the cases. These results confirm that gene sequence and expression context evolve independently after a gene duplication in 1–2 orthologous groups.

### Diverged expression contexts in the two β-subunits of the Nascent polypeptide-associated complex in S. cerevisiae

As an example, we have looked in detail at a pair of in-paralogs in *S. cerevisiae *with a large difference in expression context conservation: β_1_NAC (EGD1) and β_3_NAC (BTT1). This example was selected because the in-paralogs in *S. cerevisiae *have an especially large difference in expression context conservation relative to *C. elegans *(for this species pair, the microarray data had the highest predictive relevance of all our species comparisons; see paragraph "Correlation of sequence identity and expression context conservation between orthologous groups" and Figs. 2d-f in [6]). In general, one should be alert when interpreting microarray data for a particular gene. For example, its spot may not hybridize well and the level of expression, co-expression or even expression context of the gene will be correspondingly influenced. We therefore checked these two genes and found that they behave normally: the fraction of experiments where they are over- and under-expressed is comparable to that of average genes (not shown).

The β-subunit of the Nascent polypeptide-Associated Complex (βNAC) is represented by two copies in *S. cerevisiae*: β_1_NAC (EGD1) and β_3_NAC (BTT1) [15,16]. Other species have only one copy of this gene: icd-1 in *C. elegans*, bic in *D. melanogaster *and BTF3 in *H. sapiens*. Comparing the expression context of each of these three genes to the two *S. cerevisiae *genes revealed that for all species comparisons, the expression context of EGD1 was highly conserved, while the expression context of BTT1 had diverged (see Table 4). Compared to icd-1 in *C. elegans*, the expression context correlation of BTT1 was even negative. When we compare the sequence identity of the two genes with their single orthologs in the other three species in this study, we find indeed that BTT1 is more diverged than EGD1 in all cases (see Table 4), i.e. sequence divergence and expression context divergence are completely consistent.

The function of these two gene copies remains unclear. So far, the only difference in function found for these two genes comes from deletion experiments. Disruption of either of the *S. cerevisiae *βNAC copies yielded viable strains, that differ only in the level of GAL1 and GAL10 induction after transmission to a medium containing galactose in stead of glucose [15]. The cross bred double negative βNAC mutant showed an increase in the expression of several genes, including the GAL genes. Hu and Ronne (1994) suggested that EGD1 and BTT1 have a redundant function, but based on the diverged expression context, it is likely that the two genes are expressed under highly divergent cellular circumstances. Given the consistent hints from the differential conservation of both the expression context and the protein sequence, we predict that EGD1 is the true ortholog of icd-1, bic and BTF3.

### Correlation of sequence identity and expression context conservation within orthologous groups after multiple gene duplications

We also compared sequence conservation with expression context conservation in more expanded X-X orthologous groups, i.e. all orthologous groups with four or more genes in two species. Here, we considered sequence identity and expression context conservation consistent if they are positively correlated over all the gene pairs within an X-X orthologous group, and inconsistent when they are negatively correlated (note that carrying out this analysis on the 1–2 orthologs would give the same results as in the paragraph "Correlation of sequence identity and expression context conservation between orthologs after a single gene duplication").

**Figure 6:**
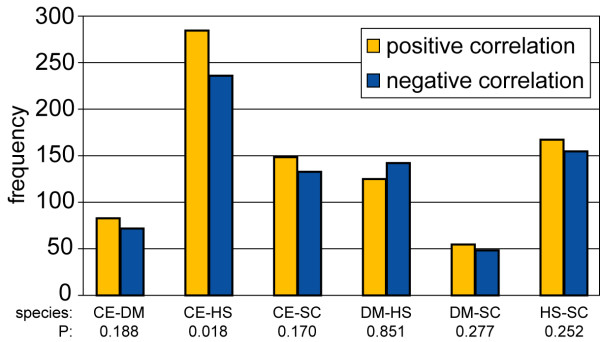
Consistency of sequence divergence with divergence in expression context for expanded orthologous groups. Consistency (positive correlation) or inconsistency (negative correlation) of sequence divergence with divergence in expression context for all expanded orthologous groups (X-X orthologs, except 1–2 orthologs). Plotted frequencies are the number of X-X orthologous groups with a positive and negative correlation. P is the probability to find at least this number of positively correlated observations by chance (binomial distribution).

Fig. 6 shows that these results and the results of the analysis of simple duplications (Fig. 5) are very comparable. In almost all species comparisons, there is no significant difference between the number of consistent and inconsistent observations (P < 0.05, binomial distribution, except CE-HS orthologs where P = 0.018). The predominantly inconsistent X-X orthologous groups between *D. melanogaster *and *H. sapiens *may be the result of the lower predictive relevance of the expression data in these species (as mentioned in the paragraph "Correlation of sequence identity and expression context conservation between orthologous groups").

If in both species the most conserved X-X orthologs are the only two genes with a selective constraint to maintain the ancestral function, the less conserved X-X orthologs may diverge randomly. Thus, it is possible that the negative correlation between sequence identity and expression context conservation in the whole X-X orthologous group arose by chance. For those X-X orthologous groups with a negative correlation, we therefore checked if there was one gene pair that harbored both the highest expression context conservation and the highest sequence identity. However, this was the case for only 10% of these inconsistent X-X orthologous groups, so we must conclude that their negative correlation between sequence identity and expression context conservation is not the result of one of the X-X orthologous gene pairs being conserved, and the rest of the genes diverging randomly. Rather, the conclusion is that as in 1–2 orthologs, the sequence and the expression context also evolve independently in other, more expanded X-X orthologous groups.

## Conclusion

In this paper, we introduce a global definition of expression context based on gene expression data. As equivalent tissues or experiments can not be assigned between distantly related species, our method uses orthologous genes to define convertible expression contexts between species. We represent the expression context of a query gene as the co-expression profile with a range of genes, rather than as the expression profile across corresponding experimental conditions. Though the microarrays were carried out under highly divergent conditions in the four Eukaryotes in this study (see Fig. 1b in [6]), the expression context of one gene is based on many expression correlation values, each of which in turn integrates a large collection of experiments. To test the coverage and homogeneity of the experimental data sets, we calculated the expression correlation values of all gene pairs separately over two random halves of the microarray experiments. In *D. melanogaster *(r = 0.91) and *S. cerevisiae *(r = 0.79), these scores were highly correlated (the correlation was not calculated for *C. elegans *and *H. sapiens *as these data sets were very large). Thus, we do not expect biases in the microarray experimental conditions to severely influence the correlations in expression context. Application of our method reveals that the expression context is conserved between orthologs across all species pairs, though X-X orthologs are less well conserved than 1-1 orthologs (see Fig. 2). We also find that informational genes have a more conserved expression context than operational genes (see Fig. 4). Taken together, these results show that the expression context presented here is a meaningful measure of the global expression context of a gene.

Using this method, we analyzed the correlation between the rates of evolution of the protein sequence and of the expression context. A correlation might be expected if the selective constraints on sequence and expression context were linked. In a comparison between all unexpanded orthologous groups, we find that this correlation is very low (see Table 3). This analysis compares genes that have branched apart at the speciation event, which means all differences in sequence conservation or expression context conservation are due to orthologous group specific evolution rates. Because of the wide range of functions carried out by the different orthologous groups, it is likely that there are also differences in the evolution rates between orthologous groups. To eliminate the possible resulting biases in the comparison between orthologous groups, we have also compared the rates of sequence and expression context evolution within orthologous groups, i.e. after one (1–2 orthologous groups) or multiple (X-X orthologous groups) gene duplication events. In these analyses, not all genes in one comparison have originated at the same time, but biases due to orthologous group specific evolution rates are absent. Still, the conclusions are the same as in the comparison between orthologous groups. For 1–2 orthologs as well as for the other X-X orthologs, the cases where sequence identity and expression context conservation were correlated were not significantly over-represented (see Figs. 5 and 6). The only species pair with significantly more consistent observations in both analyses was *C. elegans *and *H. sapiens*, though only the CE1-HS2 and not the HS1-CE2 orthologs were consistent. Comparing the types of microarray experiments carried out in these two species shows that there is little overlap [6]. Nonetheless, these species are almost the only pair with a significant over-representation of consistency between sequence identity and expression context conservation.

The methods employed in this research show that the expression context is conserved in orthologs between species. Sequence identity and expression context conservation are not correlated after gene duplication. Thus, annotation of different expression contexts to orthologs can not be based on sequence similarity alone.

Many of the expression correlations that compose the expression context may be irrelevant. According to the global definition of expression context introduced here, the expression correlation scores of all 1-1 orthologs in the genome add to the expression context. As few genes will possess a functional network containing all 1-1 orthologs, many co-expression values in the vector defining the expression context may be irrelevant. As an alternative, we have therefore also performed all analyses presented in this research using another method, that defined the expression context conservation as the number of overlapping orthologous groups in the top100 co-expressed 1-1 orthologs between two genes. In other words, this method counts how many of the highly co-expressed 1-1 orthologs are shared between two genes. Qualitatively, the results found using this alternative method were identical, indicating a robustness of the results to different definitions of expression context.

Previously, we have shown that after a gene duplication, one of the in-paralogs has a tendency to keep the ancestral regulatory interaction, while this link is lost in the other [7]. We could not find evidence for such partial conservation using the global definitions of functional conservation introduced here. In other words, although reliably predicted co-regulatory links are asymmetrically conserved after gene duplication, the co-expression of in-paralogs remains similar from a global point of view. This can be explained if the divergence (which we observe studying pairwise links) indicates sub-functionalization, while the in-paralogs remain within in the same cellular process (resulting in a similar global expression context).

## Methods

### Data

The expression correlation of more than 326 million gene pairs over a large number of DNA microarrays in *C. elegans*, *D. melanogaster*, *H. sapiens *and *S. cerevisiae *[6] was calculated using uncentered correlation (see Fig. 1a). We used this data set as is, because it is the largest uniform collection of gene expression data available for Eukaryotes. The genomes were downloaded from Wormbase for *C. elegans *[17], Flybase for *D. melanogaster *[18], Refseq for *H. sapiens *[19] and the Saccharomyces Genome Database for *S. cerevisiae *[20]. The genome of *A. gossypii *was downloaded from the Ashbya Genome Database [21].

### Similarity and orthology

We searched the genomes for homologs using the Smith-WatermanP algorithm [22] on a TimeLogic DeCypher in all query-database combinations (matrix: Blosum62; e-value cutoff:100). In the case of spurious asymmetries in the similarity search (e.g. two sequences giving different alignments depending on which was the query), the results are the average of two values, including both reciprocal experiments. Inparanoid [9] was run on the search results (default parameters; score cutoff:50; outgroup cutoff:50; sequence overlap cutoff:0.5; confidence cutoff:0.05; group overlap cutoff:0.5; gray zone:0). We only included genes in the orthology analysis if microarray data was available. For each pair of species, the 1-1 orthologous groups (one ortholog in each species, see Table 1) were used to define the expression context of a gene (see below and Fig. 1). The rest of the orthologous groups were considered gene expansions (X-X orthologous groups, with more than one ortholog in at least one of the species). There are about twice as many 1-1 orthologs as there are X-X orthologous groups (see Table 1).

### Expression context

The expression context of a gene was estimated using the co-expression values with the other genes in the genome. To be able to make an unambiguous comparison between two species, we only used the co-expression values with the 1-1 orthologs (see Fig. 1b). We only included 1-1 orthologs in the list if we had co-expression data available in both species. The expression context conservation between two genes is defined as Pearson's correlation coefficient between the two vectors with co-expression values with the 1-1 orthologs.

The expected level of consistency between the sequence identity and the expression context conservation in a completely consistent set of 1–2 orthologs was calculated by separating the expression context conservation scores into two data sets. One contained the highest expression context correlation score in each 1–2 orthologous group (most conserved 1–2 orthologs, cf. Fig. 4), the other contained the lower scores (less conserved 1–2 orthologs). We then randomly assigned the values from the high, most conserved data set to the 1–2 orthologous pairs with the highest sequence identity, and the values from the low, less conserved data set to the 1–2 orthologous pairs with the lowest sequence identity, and counted the consistent cases. Thus, all orthologous groups were consistent in principle, and inconsistent observations can result only from the overlap of the distributions of the expression context conservation scores (cf. Fig. 2). The numbers found (triangles in Fig. 5) are thus the maximum expected number of consistent observations and the minimum expected number of inconsistent observations if the data would have been completely consistent, given the overlapping distributions.

### KOG classification

The list of KOGs (euKaryotic clusters of Orthologous Groups of proteins) with assigned genes was downloaded from the COG website [10].

### K_a_/K_s _ratio

The K_a_/K_s _ratio was calculated using the kaks function of the seqinr package of the R Project for Statistical Computing [23]. This function makes an unbiased estimate of the ratio of nonsynonymous (K_a_) to synonymous (K_s_) nucleotide substitution for a set of aligned sequences [24].

## Authors' contributions

BED carried out the analyses, participated in the design and drafted the manuscript. MAH and BS conceived of the study, participated in the design and coordination and helped to draft the manuscript.

## Figures and Tables

**Table 1:** Inparanoid pairwise orthologous groups between all species pairs for *C. elegans *(15950 genes) *D. melanogaster *(4456 genes) *H. sapiens *(12193 genes) and *S. cerevisiae *(6199 genes).

**species A**	**species B**	**total OGs**	**1-1 OGs**
*C. elegans*	*D. melanogaster*	2393	1907
*C. elegans*	*H. sapiens*	3814	2335
*C. elegans*	*S. cerevisiae*	2520	1516
*D. melanogaster*	*H. sapiens*	2739	1891
*D. melanogaster*	*S. cerevisiae*	1641	1193
*H. sapiens*	*S. cerevisiae*	2514	1580
total		15621	10422

**Table 2:** Probability that the expression context conservation scores in different classes of orthologs and random non-orthologous gene pairs were drawn from the same distribution (see histograms in Fig. 2; Pvalues, Student's t-test; the distributions are normal according to a Shapiro-Wilk test, P < 1·10^-4^). The expression context data is combined over all species comparisons: 1-1 orthologs (n = 10303) all X-X orthologs (n = 27147) most conserved X-X orthologs (n = 5180) less conserved X-X orthologs (n = 21967) random non-orthologous gene pairs (n = 6000).

	**1-1 orth**	**most cons X-X**	**less cons X-X**	**random non-orth**
**all X-X orth**	6.31·10^-233^	0	1.78·10^-70^	3.55·10^-21^
**random non-orth**	9.66·10^-173^	0	0.172	.
**less cons X-X**	0	0	.	.
**most cons X-X**	1.38·10^-57^	.	.	.

**Table 3:** Correlation between sequence identity and expression context conservation for 1-1 orthologs between all species pairs. P is the probability that the data set is a sample drawn from a distribution with correlation coefficient zero.

**species A**	**species B**	**correlation**	**P**
*C. elegans*	*D. melanogaster*	0.077	8.41·10^-4^
*C. elegans*	*H. sapiens*	0.060	4.49·10^-3^
*C. elegans*	*S. cerevisiae*	0.121	5.14·10^-6^
*D. melanogaster*	*H. sapiens*	0.092	6.27·10^-5^
*D. melanogaster*	*S. cerevisiae*	0.050	9.01·10^-2^
*H. sapiens*	*S. cerevisiae*	0.061	1.46·10^-2^

**Table 4:** Sequence identity and expression context conservation of the two βNAC in-paralogs in *S. cerevisiae*. The β subunit of the Nascent polypeptide-Associated Complex has two orthologs in *S. cerevisiae*: Enhanced Gal4 DNA binding protein 1 (EGD1, β_1_NAC) and Basic Transcription factor Three 1 (BTT1, β_3_NAC). The three other species in this analysis have only one ortholog: inhibitor of cell death 1 (icd-1 in *C. elegans*), bicaudal (bic in *D. melanogaster*) and Basic Transcription Factor 3 (BTF3 in *H. sapiens*).

		***C. elegans *icd-1**	***D. melanogaster *bic**	***H. sapiens *BTF3**
***S. cerevisiae *EGD1**	**identity**	0.385	0.350	0.375
***S. cerevisiae *EGD1**	**exp. cont.**	0.302	0.203	0.199
***S. cerevisiae *BTT1**	**identity**	0.300	0.305	0.340
***S. cerevisiae *BTT1**	**exp. cont.**	-0.205	-0.092	0.006
